# Cytotype diversity and genome size variation in *Knautia* (Caprifoliaceae, Dipsacoideae)

**DOI:** 10.1186/s12862-015-0425-y

**Published:** 2015-07-17

**Authors:** Božo Frajman, Ivana Rešetnik, Hanna Weiss-Schneeweiss, Friedrich Ehrendorfer, Peter Schönswetter

**Affiliations:** Institute of Botany, University of Innsbruck, Sternwartestraße 15, A-6020 Innsbruck, Austria; Faculty of Science, University of Zagreb, Marulićev trg 20/II, HR-10000 Zagreb, Croatia; Department of Botany and Biodiversity Research, University of Vienna, Rennweg 14, A-1030 Vienna, Austria

**Keywords:** Chromosome numbers, Cytotype diversity, Genome downsizing, Genome size, *Knautia*, Polyploidy

## Abstract

**Background:**

Polyploidisation is one of the most important mechanisms in the evolution of angiosperms. As in many other genera, formation of polyploids has significantly contributed to diversification and radiation of *Knautia* (Caprifoliaceae, Dipsacoideae). Comprehensive studies of fine- and broad-scale patterns of ploidy and genome size (GS) variation are, however, still limited to relatively few genera and little is known about the geographic distribution of ploidy levels within these genera. Here, we explore ploidy and GS variation in *Knautia* based on a near-complete taxonomic and comprehensive geographic sampling.

**Results:**

Genome size is a reliable indicator of ploidy level in *Knautia*, even if monoploid genome downsizing is observed in the polyploid cytotypes. Twenty-four species studied are diploid, 16 tetraploid and two hexaploid, whereas ten species possess two, and two species possess three ploidy levels. Di- and tetraploids are distributed across most of the distribution area of *Knautia*, while hexaploids were sampled in the Balkan and Iberian Peninsulas and the Alps.

**Conclusions:**

We show that the frequency of polyploidisation is unevenly distributed in *Knautia* both in a geographic and phylogenetic context. Monoploid GS varies considerably among three evolutionary lineages (sections) of *Knautia*, but also within sections *Trichera* and *Tricheroides*, as well as within some of the species. Although the exact causes of this variation remain elusive, we demonstrate that monoploid GS increases significantly towards the limits of the genus’ distribution.

**Electronic supplementary material:**

The online version of this article (doi:10.1186/s12862-015-0425-y) contains supplementary material, which is available to authorized users.

## Background

The evolution of almost all angiosperm lineages is characterised by numerous polyploidisation events [1] that significantly contributed to their diversification and radiation [2–4]. Polyploidisation can directly influence gene flow within and among taxa [5] and often confers instantaneous speciation [6]. On long-terms it may govern habitat preferences [7] and consequently bear on the distribution of taxa [6]. The effects of polyploidisation are, however, far from uniform and may have an impact on species morphology, physiology and interactions with the environment [8]. Therefore, knowledge of the ploidy level variation within and among taxa is of principal importance for understanding the evolutionary pathways in any plant group and facilitates the interpretation of phylogenetic relationships.

The nuclear DNA content may vary considerably among closely related species but is remarkably constant within most species [9–11], thus serving as an important taxonomic character [8]. For some species, however, significant variation among populations of the same ploidy level has been demonstrated, which is often geographically structured [12–14]. In addition, it has been shown that several ploidy level cytotypes (for simplicity, in the following we restrict the term “cytotype” to ploidy levels) exist within some species [12–16], and such intraspecific polyploids often have multiple origins [17]. The genome size (GS) of polyploids is expected to increase in direct proportion with ploidy level in recently formed polyploid series, whereas in older polyploids a significant decrease of monoploid GS, termed genome downsizing [18], may take place [14, 19–21]. Increase of monoploid GS in polyploids in comparison to diploid progenitors is less well documented and probably less common (e.g., *Hordeum*, [22]; *Nicotiana*, [23]; *Melampodium*, [24]).

Flow cytometry (FCM; see [8] and references therein), a high-throughput technique for the estimation of DNA content, has become an essential tool in evolutionary and systematic research in vascular plants. In recent years its large-scale application has been pivotal for elucidating polyploid speciation events, infraspecific ploidy variation and ecological differentiation of cytotypes; furthermore, flow cytometric data have supplemented phylogenetic evidence [12, 14, 25–27]. Nevertheless, the understanding of fine and broad scale patterns of ploidy variation is limited to relatively few species, and studies exploring variation in ploidy level and/or GS within taxa from different geographic areas are much needed [28], as are studies covering entire species-rich genera. Detailed cytogeographical studies at the genus level are rare (but see, [29–31]), whereas numerous studies have focussed on particular polyploid species complexes [7, 12–14, 32].

The genus *Knautia* L. (Caprifoliaceae, Dipsacoideae) comprises 50–55 species distributed in western Eurasia and northwestern Africa. The highest species diversity is in southern and southeastern Europe, especially the Alps and the Balkan Peninsula. *Knautia* species inhabit dry grasslands, wet meadows, alpine grasslands, forests, and ruderal communities [33, 34]. The traditional division of *Knautia* into the mostly perennial sect. *Trichera* encompassing the majority of taxa as well as the annual, species-poor sections sect. *Tricheroides* (*K. byzantina*, *K. integrifolia*) and sect. *Knautia* (*K. orientalis*; [35]) has recently been confirmed phylogenetically, but the position of *K. degenii* was ambiguous, either in sect. *Knautia* or sect. *Tricheroides* [34]. The three sections are characterised by karyotypes A, B and C, respectively, which differ in basic chromosome number and/or karyotype structure [35–37]. The perennial sect. *Trichera* is characterized by *x* = 10 and karyotype A, with ploidy levels ranging from diploids via tetraploids to hexaploids [35, 36]. In contrast, the species in the annual sections are exclusively diploid with *x* = 10 (sect. *Tricheroides*, karyotype B) and *x* = 8 (sect. *Knautia*, karyotype C). They are restricted to the eastern Balkan Peninsula and northwestern Anatolia; an exception is *K. integrifolia*, which extends its range to the Iberian Peninsula [36].

Sect. *Trichera* is characterized by the lack of clear and reliable morphological differential characters and a high incidence of hybridisation [34, 36]; it qualifies as a text-book example for a taxonomically difficult group. In the most comprehensive treatment of sect. *Trichera* based on morphology, distribution patterns and ploidy level, Ehrendorfer [35] has placed all taxa into eleven informal groups, most of which were recently shown to be non-monophyletic ([34], Frajman *et al.*, unpubl. res.). Ehrendorfer [35] demonstrated that several species contain multiple ploidy levels (e.g., di- and tetraploid *K. arvensis* and *K. drymeia*, or tetra- and hexaploid *K. dipsacifolia*), but none of the species includes all three ploidy levels. This was also confirmed by subsequent karyological studies [38–43]. Recently, Kolář *et al.* [44] used flow cytometry to investigate patterns of ploidy variation in *K. arvensis* in Central Europe, and Temsch and Greilhuber [45] as well as Siljak-Yakovlev *et al.* [46] provided GS values for eight taxa and one taxon, respectively.

The aim of the present study is to explore GS variation and the incidence of polyploidy within the genus *Knautia* based on a comprehensive taxonomic and geographic sampling of 381 populations of 54 *Knautia* species, mostly from sect. *Trichera*. Our specific aims were (1) to screen ploidy levels and to estimate GS in order to determine geographical patterns of cytotype distributions as well as to identify populations and species with multiple ploidy levels; (2) to test previously published karyological records and to determine chromosome numbers for taxa with uncertain ploidy level; (3) to prove that GS allows reliable inference of ploidy levels in *Knautia* by using a combination of flow cytometry and chromosome number estimation in several taxa, (4) to test if genome downsizing is associated with polyploid genome evolution in *Knautia*, and (5) to explore how GS evolved in different phylogenetic lineages of diploid *Knautia*.

## Results

### Chromosome numbers

**Fig. 1:**
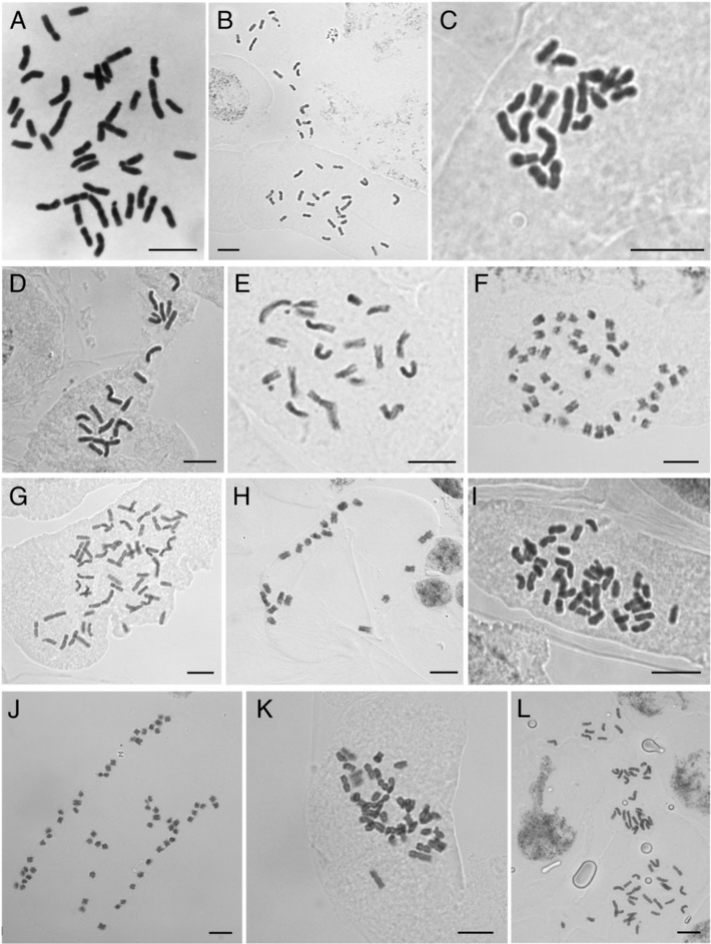
Mitotic chromosomes of *Knautia* species. **a** *K. arvensis* K455, 2*n* = 4*x* = 40. **b** *K. arvernensis* K387, 2*n* = 4*x* = 40. **c** *K. basaltica* K386, 2*n* = 2*x* = 20. **d** *K. collina* K414, 2*n* = 2*x* = 20. **e** *K. drymeia* K312, 2*n* = 2*x* = 20. **f** *K. foreziensis* K380, 2*n* = 4*x* = 40 + 1B. **g** *K. illyrica* K045, 2*n* = 6*x* = 60. **h** *K. mollis* K373, 2*n* = 2*x* = 20. **i** *K. nevadensis* K406, 2*n* = 4*x* = 40. **j** *K. ressmannii* K450, 2*n* = 6*x* = 60. **k** *K. rupicola* K400, 2*n* = 4*x* = 40. **l** *K. rupicola* K402, 2*n* = 6*x* = 60. Details for the sampling localities are given in the Additional file 2: Table S1

Chromosome numbers were established for 15 individuals from 14 *Knautia* species (Table 1, Fig. 1). Three ploidy levels were recorded, diploids (2*n* = 2*x* = 20), tetraploids (2*n* = 4*x* = 40) and hexaploids (2*n* = 6*x* = 60). One species (*K. rupicola*) was found to possess two ploidy levels. In *K. foreziensis* (Fig. 1f) an additional chromosome was observed, which likely represents a B-chromosome but more detailed analyses have to be performed to exclude aneuploidy. Cut-out karyotypes were prepared for six individuals representing six species, three of which are diploid, two tetraploid and one hexaploid (Additional file 1: Figure S1). The karyotypes cannot be directly compared as it is impossible to define homo- or homeologous chromosome pairs based on chromosome morphology alone. Karyotypes of diploid species are composed mostly of meta-, submeta- to acrocentric chromosomes, one or two of which carry visible NOR regions, usually as subterminal satellites (Additional file 1: Figure S1). Polyploids have similar types of karyotypes, but the low number of chromosome slides available to prepare karyotypes prevented direct comparisons.

**Table 1:** Summary results of flow cytometric analyses in Knautia, using DAPI (relative DNA content, given in arbitrary units) and PI (absolute DNA content, given in pg) fluorochromes as well as the determined chromosome number

Taxon	Section	Estimated ploidy level	Relative genome size (mean)	Intracytotype variation, max/min (%)	Mean monoploid relative genome size	RGS N measured individuals/populations	Absolute genome size (pg)	AGS N measured individuals/populations	Chromosome number (ID)
*K. adriatica* Ehrend.	*Trichera*	4*x*	0.579		0.145	5/1			
*K. albanica* Briq.	*Trichera*	2*x*	0.286–0.291 (0.288)	1.75	0.144	20/2	7.26–7.64	2/1	
*K. ambigua* Boiss. & Orph	*Trichera*	2*x*	0.279–0.300 (0.292)	7.53	0.146	32/7			
*K. arvensis* (L.) Coult.	*Trichera*	2*x*	0.277–0.305 (0.291)	10.11	0.145	184/33			
		4*x*	0.551–0.585 (0.567)	6.17	0.142	68/14	13.93–14.11	2/1	40 (K455)
*K. arvensis × kitaibelii*	*Trichera*	4*x*	0.546–0.587 (0.566)	7.51	0.142	10/2			
*K. arvernensis* (Briq.) Szabó	*Trichera*	4*x*	0.575–0.595 (0.586)	3.48	0.147	30/6	14.84–15.15	4/2	40 (K387)
*K. baldensis* Kern. & Szabó	*Trichera*	4*x*	0.550–0.563 (0.556)	2.36	0.140	32/5	14.36–14.79	2/1	
*K. basaltica* Chass. & Szabó	*Trichera*	2*x*	0.306–0.309 (0.308)	0.98	0.154	10/2	7.51–7.72	4/2	20 (K386)
*K. calycina* (Presl) Guss.	*Trichera*	2*x*	0.305–0.341 (0.318)	11.80	0.159	111/14	8.00–8.29	2/1	20 (K323)
*K. carinthiaca* Ehrend.	*Trichera*	2*x*	0.269–0.276 (0.273)	2.60	0.137	20/2			
*K. clementii* (Beck) Ehrend.	*Trichera*	4*x*	0.528–0.547 (0.540)	3.60	0.135	20/3	14.64	1/1	
	*Trichera*	6*x*	0.761		0.127	5/1			
*K. collina* (Req. ex Guérin) Jord.	*Trichera*	2*x*	0.279–0.336 (0.323)	20.43	0.162	36/8	8.58–8.80	4/2	20 (K414)
*K. csikii* Jáv. & Szabó	*Trichera*	2*x*	0.282–0.293 (0.287)	3.90	0.144	10/2			
*K. dalmatica* Beck	*Trichera*	4*x*	0,551*		0.138*	5/1			
*K.* cf *degenii* Borbás	*Tricheroides*	2*x*	0.242–0.244 (0.243)	0.83	0.122	7/2			
*K. dinarica* K. Malý	*Trichera*	2*x*	0.258–0.283 (0.267)	9.69	0.134	62/9			
		4*x*	0.494–0.58 (0.536)	17.41	0.135	112/16	13.57–14.11	5/4	
*K. dipsacifolia* Kreutz.	*Trichera*	4*x*	0.522–0.56 (0.537)	7.28	0.134	25/3			
		6*x*	0.782–0.856 (0.824)	9.46	0.137	77/11	20.81–21.57	4/2	60 (K029)
*K. drymeia* Heuff.	*Trichera*	2*x*	0.251–0.303 (0.281)	20.72	0.141	136/25	7.27–7.44	6/4	20 (K312)
		4*x*	0.495–0.582 (0.547)	17.58	0.137	198/39			
*K. fleischmannii* (Hladn. ex Rchb.) Pach.	*Trichera*	4*x*	0.583		0.146	10/1			
*K. foreziensis* Chass. & Szabó	*Trichera*	4*x*	0.596		0.149	9/2	14.80–15.30	2/1	40 + 1B (K380)
*K. godetii* Reut.	*Trichera*	2*x*	0.296–0.303 (0.299)	2.36	0.150	15/3			
*K. illyrica* Beck	*Trichera*	2*x*	0.286–0.298 (0.292)	4.20	0.146	25/3			
		4*x*	0.554–0.593 (0.573)	7.04	0.143	40/6			
		6*x*	0.877		0.146	10/1	21.13–21.25	2/1	60 (K045)
*K. integrifolia* (L.) Bertol.	*Tricheroides*	2*x*	0.120–0.192 (0.164)	60.00	0.082	109/21	3.90–4.11	2/1	
*K. involucrata* Sommier & Levier	*Trichera*	2*x*	0.36		0.180	5/1			
*K. kitaibelii* (Schult.) Borb.	*Trichera*	4*x*	0.531–0.554 (0.543)	4.33	0.136	6/2			
*K. lebrunii* J. Prudhomme	*Trichera*	2*x*	0.301–0.306 (0.304)	1.66	0.152	10/2	7.86–8.08	3/2	
*K. legionensis* DC.	*Trichera*	4*x*	0.633–0.643 (0.637)	1.58	0.159	15/3	16.26–16.76	4/2	
*K. longifolia* (Waldst. & Kit.) Koch	*Trichera*	2*x*	0.268–0.293 (0.281)	9.33	0.141	68/7	7.05–7.24	5/3	
*K. lucana* Lacaita & Szabó	*Trichera*	2*x*	0.31		0.155	9/1	7.57	1/1	
*K. macedonica* Griseb.	*Trichera*	2*x*	0.281–0.295 (0.290)	4.98	0.145	18/3			
*K. magnifica* Boiss. & Orph.	*Trichera*	4*x*	0.505–0.551 (0.524)	9.11	0.131	28/4			
*K. midzorensis* Form.	*Trichera*	2*x*	0.276–0.281 (0.279)	1.81	0.140	20/3			
		4*x*	0.500–0.544 (0.527)	8.80	0.132	41/8			
*K. mollis* Jord.	*Trichera*	2*x*	0.328–0.330 (0.329)	0.61	0.165	10/2	8.23–8.58	4/2	20 (K373)
*K. montana* (Bieb.) D.C.	*Trichera*	2*x*	0.332–0.360 (0.344)	8.43	0.172	18/4			
*K. nevadensis* Szabó	*Trichera*	4*x*	0.600–0.645 (0.624)	7.50	0.156	20/4	15.65–16.92	3/2	40 (K405; K406)
*K. norica* Ehrend.	*Trichera*	4*x*	0.540–0.549 (0.545)	1.67	0.136	23/4			
*K. orientalis* L.	*Knautia*	2*x*	0.353–0.368 (0.360)	4.25	0.180	20/4			
*K. pancicii* Szabó	*Trichera*	2*x*	0.273		0.137	9/1			
*K. pectinata* Ehrend.	*Trichera*	2*x*	0.288		0.144	10/1			
		4*x*	0.556–0.586 (0.570)	5.40	0.143	30/4	15.00	1/1	40 (K100)
*K. persicina* Kern.	*Trichera*	4*x*	0.562–0.582 (0.572)	3.56	0.143	20/2	14.87–15.54	2/1	
*K. purpurea* (Vill.) Borb.	*Trichera*	2*x*	0.299–0.312 (0.305)	4.35	0.153	20/3	7.70–8.59	4/2	
		4*x*	0.576–0.577 (0.576)	0.17	0.144	15/2			
*K. ressmannii* (Pach.) Briq.	*Trichera*	6*x*	0.841–0.866 (0.851)	2.97	0.142	12/3	21.49–21.67	4/3	60 (K448, K450)
*K. rupicola* (Willk.) Font Quer	*Trichera*	4*x*	0,730		0.183	5/1	23.09	1/1	40 (K400)
	*Trichera*	6*x*	1.057–1.063 (1.060)	0.57	0.177	6/2	25.49–26.33	2/1	60 (K402)
*K. sarajevensis* Szabó	*Trichera*	4*x*	0.465–0.544 (0.513)	16.99	0.128	65/8	13.66–13.78	2/1	
*K. slovaca* Stepanek	*Trichera*	2*x*	0.280–0.292 (0.286)	4.29	0.143	7/3			
*K. subcanescens* Jord.	*Trichera*	2*x*	0.288–0.290 (0.289)	0.69	0.145	10/2	7.44–7.61	3/2	
	*Trichera*	4*x*	0.582		0.146	5/1			
*K. subscaposa* Boiss. & Reut.	*Trichera*	2*x*	0.411–0.424 (0.417)	3.16	0.209	28/6			
*K. travnicensis* Beck	*Trichera*	2*x*	0.263–0.296 (0.282)	12.55	0.141	13/3			
		4*x*	0.526		0.132	10/1			
		6*x*	0.759–0.802 (0.778)	5.67	0.130	29/4			
*K. velebitica* Szabó	*Trichera*	2*x*	0.296–0.318 (0.307)	7.43	0.154	35/4	7.49	1/1	
*K. velutina* Briq.	*Trichera*	2*x*	0.292–0.309 (0.302)	5.82	0.151	40/6	7.61–7.81	2/1	
*K. visianii* Szabó	*Trichera*	2*x*	0.285–0.304 (0.293)	6.67	0.147	87/13	7.29–7.61	4/2	
*K. wagneri* Briq.	*Trichera*	4*x*	0.488		0.122	3/1			
*K.* sp.1	*Trichera*	6*x*	0.775–0.828 (0.796)	6.84	0.133	32/4			
*K.* sp.2	*Trichera*	4*x*	0.51		0.128	5/1			

* samples with low quality of RGS, used only to determine ploidy level but not included in statistical analyses

### Flow cytometry and estimations of DNA-ploidy levels

Relative DNA content (RGS) was measured in 2253 individuals from 381 populations of 54 *Knautia* species, and absolute genome size (AGS) was determined for 88 individuals from 52 populations of 30 species (Table 1, Additional file 2: Table S1). In nine populations RGS measurements were of low quality (CV exceeding the threshold of 5 %) and were thus excluded from the statistical analyses and only used for ploidy level determination. The cytometrically determined ploidy level corresponded to the estimated chromosome numbers in all karyologically analysed samples.

**Fig. 2:**
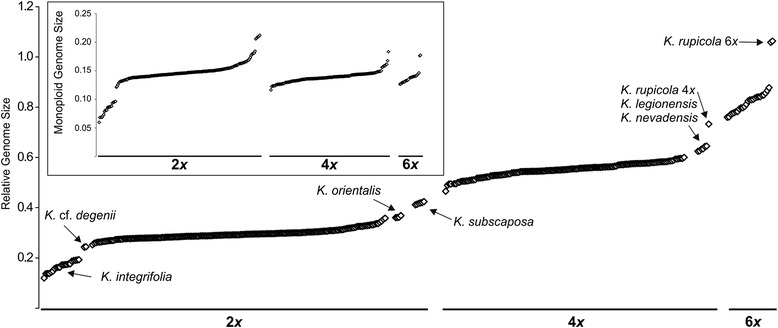
Genome size variation in *Knautia*: relative genome size, monoploid relative genome size (insert). Taxa mentioned in the Discussion are indicated

The correlation between mean AGS and RGS obtained from the same populations was high across the whole GS range (r = 0.969, *p* = 0.01) and across diploids and tetraploids (diploids r = 0.858; tetraploids r = 0.729; *p* = 0.000), but not hexaploids (r = 0.536, *p* = 0.215), which is likely explained by the fact that AGS was determined for only 12 hexaploid individuals. The RGS ranged from 0.120 in diploid *K. integrifolia* (K226) to 1.063 in hexaploid *K. rupicola* (K402), exhibiting 8.86-fold variation (Table 1, Fig. 2), whereas the AGS varied between 3.90 pg in *K. integrifolia* (K314) and 26.33 pg in *K. rupicola* (K402), exhibiting 6.75-fold variation. According to RGS all analysed populations were separated into three ploidy levels (only populations with a single ploidy level were observed): DNA-diploids (52.6 %), DNA-tetraploids (40.5 %), and DNA-hexaploids (6.9 %). We use the prefix ‘DNA-’ in order to acknowledge that for most populations analysed no chromosome counts are available [47]. For simplicity, the prefix is omitted hereafter.

**Fig. 3:**
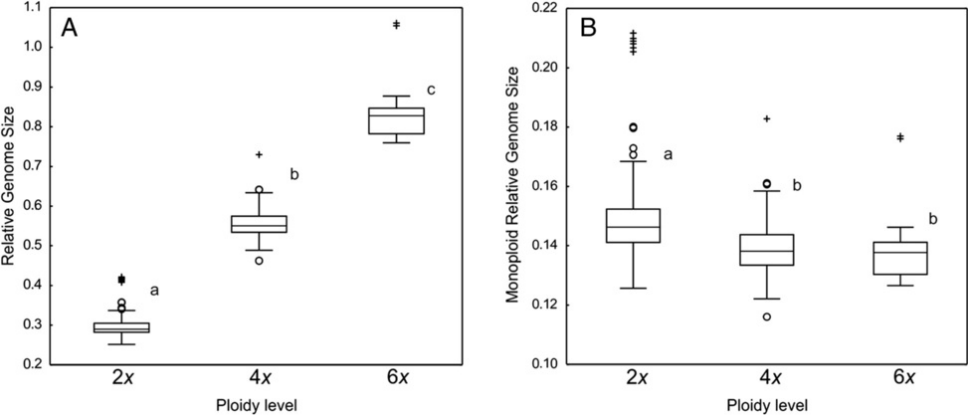
Relative genome size (**a**) and monoploid relative genome size (**b**) of cytotypes in *Knautia* sect. *Trichera*. Boxes define 25 and 75 percentiles; horizontal lines indicate medians, whiskers are from 5 to 95 percentiles, circles indicate outliers and crosses extreme values (outside the 3 box length range from the upper and lower value of the box). Means not significantly different at *P* ≤ 0.01 are indicated by the same letter (Kruskal-Wallis test followed by Mann–Whitney pairwise comparisons and Bonferroni corrections of p values)

**Fig. 4:**
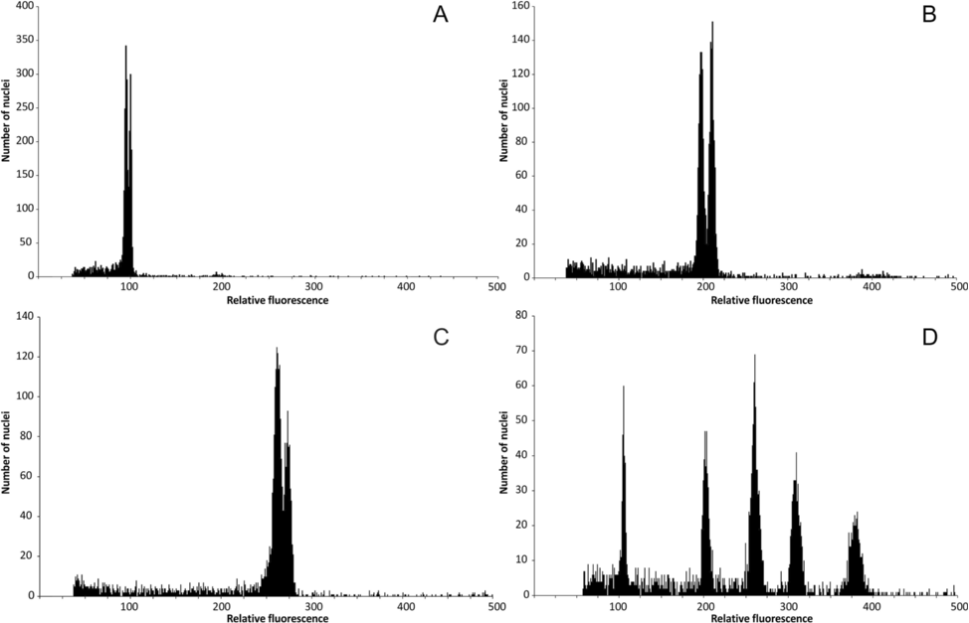
Flow cytometry histograms showing genuine differences in relative genome size. Histograms showing differences within ploidy levels of *Knautia* (**a–b**) and the divergent relative genome size of the Iberian taxa, exemplified by tetra- and hexaploid *K. rupicola* (**d**). **a** diploid *Knautia drymeia* (K040, right) and *K. lebrunii* (K408, left). **b** Tetraploid *K. nevadensis* (K405, right, and K406, left). **c** Hexaploid *K. dipsacifolia* (K029, right, and K038, left). **d** From left to right: diploid *K. drymeia* (K039), tetraploid *K. dinarica* (K028) and *K. rupicola* (K400), hexaploid *K. ressmannii* (K449) and *K. rupicola* (K402)

Forty-two out of 54 analysed taxa (77.8 %) possessed only one ploidy level, of which 57.1 % were diploids, 38.1 % tetraploids and 4.8 % hexaploids. Ten taxa (18.5 %) had two ploidy levels, of these 70 % were di-/tetraploids and 30 % tetra-/hexaploids. Two taxa (3.7 %), i.e. *K. illyrica* and *K. travnicensis*, contained all three ploidy levels. The mean RGS differed significantly between any two ploidy levels (Kruskal-Wallis test followed by Mann–Whitney pairwise comparison and Bonferroni corrections of p values; Fig. 3). Diploid populations also differed significantly from tetraploids and hexaploids in monoploid RGS, whereas there was no significant difference between tetraploids and hexaploids (Fig. 3). The same results were obtained after the exclusion of the Iberian taxa *K. legionensis*, *K. nevadensis*, *K. rupicola* and *K. subscaposa* with clearly higher monoploid RGS. Intra-ploidy variation in RGS was relatively large in all three ploidy levels in section *Trichera* (1.69-fold variation in diploids, 1.57-fold variation in tetraploids, 1.40-fold variation in hexaploids). The highest intraspecific RGS variation within a ploidy level was observed in *K. integrifolia* from sect. *Tricheroides* (1.60-fold variation); in sect. *Trichera* the highest variation was in diploid *K. drymeia* (1.21-fold variation). Intraspecific intra-cytotype variation was confirmed with simultaneous analyses of samples with different RGS, yielding histograms with one bifurcate (Fig. 4a) or two separate peaks (Figs. 4b, 4c).

### Evolution of RGS in *Knautia*

**Fig. 5:**
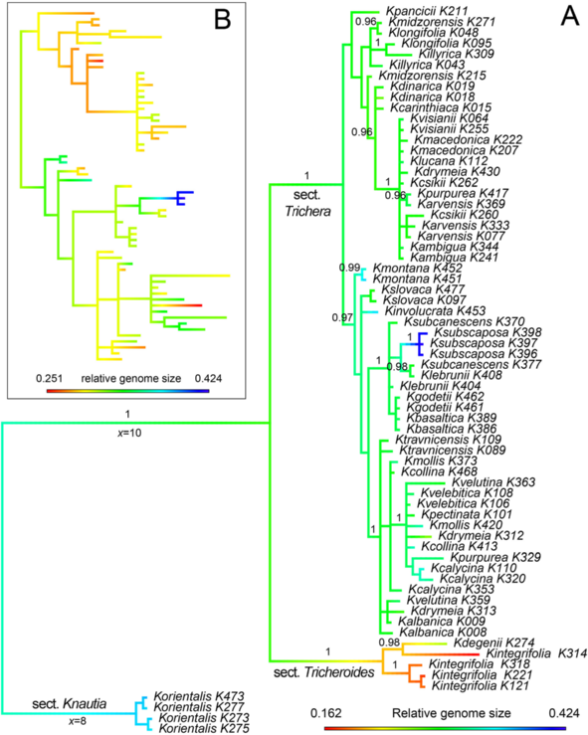
Reconstruction of relative genome size evolution in diploid members of *Knautia*. Bayesian phylogram of concatenated plastid *petN*(*ycf6*)-*psbM* and ITS sequences of (**a**) *Knautia* and (**b**) sect. *Trichera*. Reconstruction of RGS is indicated by branch colour. Values above branches in (**a**) are posterior probabilities > 0.90 derived from Bayesian analysis

Reconstruction of RGS evolution on the combined ITS and plastid phylogeny revealed a gradual increase of RGS along the branch leading to sect. *Knautia* and a decrease along the branch of sect. *Tricheroides* (Fig. 5). No such trends were observed along the branch leading to sect *Trichera*, but within this section both increasing and decreasing RGS were observed, the most striking being the increase along the branch leading to Iberian *K. subscaposa* with strongly deviating GS (Fig. 5b). The estimate of λ was 0.999, indicating that RGS exhibits a strong phylogenetic signal in *Knautia*, which was also confirmed by a Chi-square test.

### Distribution patterns of cytotypes and GS variants in sect. *Trichera*

**Fig. 6:**
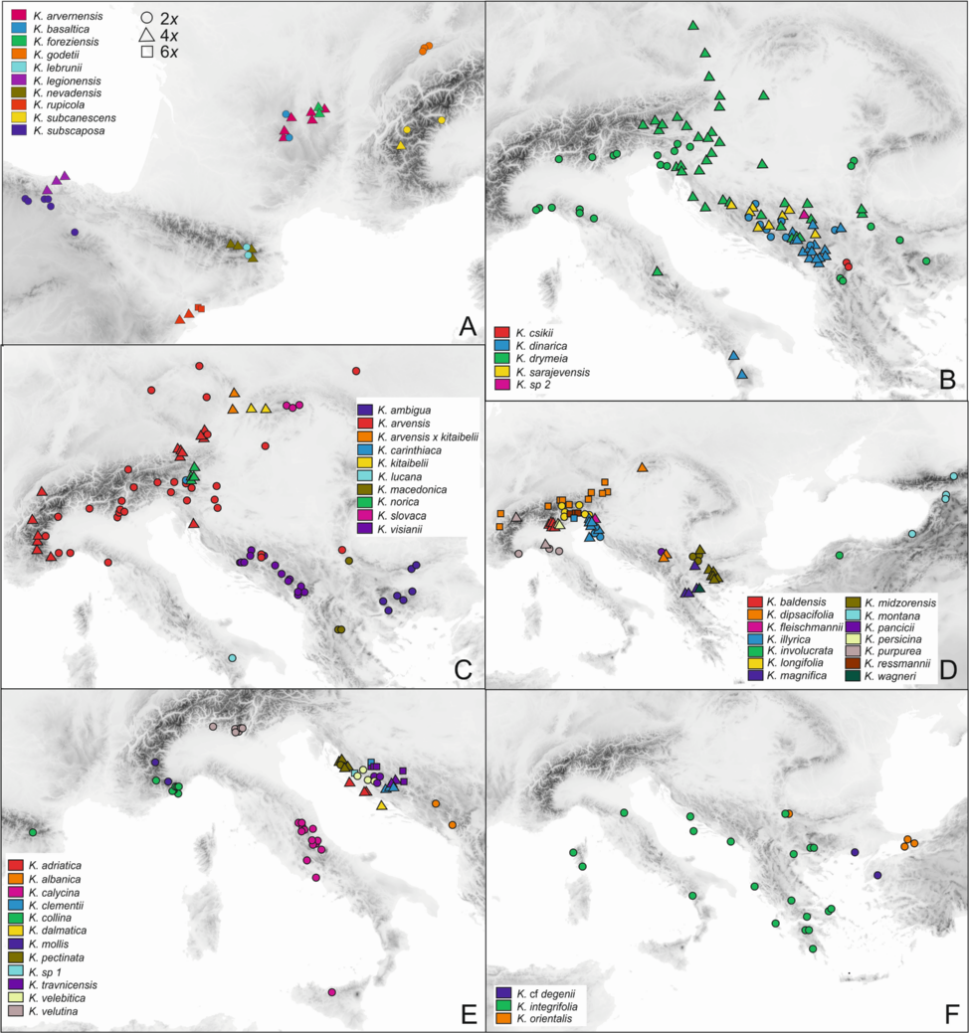
Geographical distribution of cytotypes in the genus *Knautia*. Circles: diploids; triangles: tetraploids; squares: hexaploids; colors denote taxa according to the individual legends inside each panel.

**Fig. 7:**
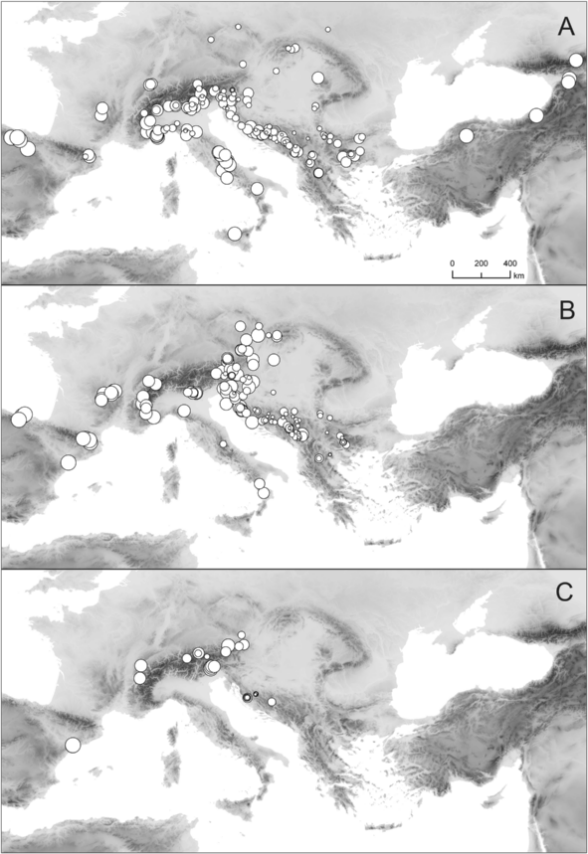
Geographical distribution of monoploid relative genome size in *Knautia* sect. *Trichera.* **a** diploids, **b** tetraploid, and **c** hexaploids. The size of a dot is proportional to the mean relative genome size of a population

Diploid and tetraploid populations of different species occur across most of the distribution area of *Knautia*, whereas hexaploid populations exhibit a more restricted distribution in the Balkans (central Dinaric Mountains), the Alps (extra-Alpine populations of *K. dipsacifolia* were not sampled) and the Iberian Peninsula (two populations of *K. rupicola* in eastern Spain; Figs. 6, 7). The monoploid RGS of di- and tetraploid individuals is smaller in the western Balkan Peninsula and the Alps and increases towards the eastern, western and southern margins of the genus’ distribution area in diploids, and towards the western and northern margins in tetraploids and hexaploids (r_s_ = 0.405, r_s_ = 0.656, and r_s_ = 0.671 *p* < 0.01 for diploids, tetraploids and hexaploids, respectively; Fig. 7).

Among the widely distributed heteroploid species (i.e., species with different ploidy levels) only *K. dinarica, K. drymeia*, *K. dipsacifolia*, and to some extent *K. arvensis* exhibit geographic patterns of cytotype distributions. (1) The northern populations of *K. dinarica* are diploid, the southern tetraploid; (2) diploid *K. drymeia* is restricted to the west and the east of the total distribution area, whereas tetraploids occur in intermittent areas and north of the diploids; (3) hexaploid *K. dipsacifolia* is limited to the Alps, whereas tetraploids occur in the Carpathians and the Balkans, (4) diploids of *K. arvensis* are mostly distributed in the inner part of the Alpine arch, whereas tetraploids extend from its outer margin towards the northeast and southeast (Fig. 6).

## Discussion

It has been acknowledged that polyploidisation has played an important role in the diversification of *Knautia* sect. *Trichera* [12, 35, 36, 42, 44, 45], but our comprehensive taxonomic and geographic sampling provides unprecedented resolution and reveals patterns not documented previously.

### Ploidy levels and chromosome numbers in *Knautia*

GS measurements with confirmatory chromosome numbers (Table 1, Figs. 1, 2, 3, 6) reveal the presence of three even ploidy levels (diploid, tetraploid and hexaploid) in *Knautia.* Minority odd ploidies (triploid, pentaploid), occasionally documented in this genus [12], were not observed in the present study. The RGS values are discretely distributed in three ranges (Figs. 2–3), fully correlated with chromosome numbers counts for 14 taxa (Fig. 1) and correspond to three ploidy levels (Table 1). The only exceptions are the Iberian taxa *K. legionensis*, *K. nevadensis*, *K. rupicola* and *K. subscaposa*, which have deviating GS (see below; Figs. 2 & 7), but their ploidy level was confirmed by chromosome numbers counted for *K. rupicola* (Fig. 1). Thus, the GS is a reliable indicator of ploidy level in *Knautia*. A similar correlation has been reported for other genera [13, 16, 48–50], but was not observed in *Cerastium* [14] or some species in Orobanchaceae [51].

In accordance with previous evidence [35, 36, 42] a high level of ploidy diversity was observed within sect. *Trichera*, whereas all members of the species-poor sections *Tricheroides* and *Knautia* are diploid [35]. Among 51 investigated taxa from sect. *Trichera*, 39 taxa (76.5 %) had a single ploidy level (diploid, tetraploid or hexaploid), ten taxa (19.6 %) had two ploidy levels (seven taxa with diploid and tetraploid cytotypes, three taxa with tetraploid and hexaploid cytotypes), and two taxa (3.9 %) included all three cytotypes (Table 1, Fig. 2). Chromosome numbers for *K. rupicola* (2*n* = 4*x* = 40 and 2*n* = 6*x* = 60; Table 1), were obtained for the first time [52] and include the only hexaploid cytotype of *Knautia* known from the Iberian Peninsula. The same applies to the chromosome numbers for hexaploid *K. illyrica* (2*n* = 6*x* = 60; previously considered exclusively tetraploid) and for tetraploid *K. pectinata* (2*n* = 4*x* = 40; previously considered exclusively diploid) (Table 1). Additionally, RGS data revealed the presence of diploidy in *K. csikii, K.* cf. *degenii*, *K. involucrata*, *K. lucana*, *K. macedonica* and *K. pancicii*, and tetraploidy in *K. legionensis* and *K. wagneri*, whose ploidy levels were previously unknown. Furthermore, in several taxa new cytotypes were found: diploids in *K. subcanescens* (previously considered uniformly tetraploid), diploids and tetraploids in *K. travnicensis* (considered hexaploid), diploids and hexaploids in *K. illyrica* (considered tetraploid) and hexaploids in *K. clementii* (considered tetraploid). On the other hand, some previously recorded cytotypes were not observed: we found only diploid populations of *K. collina*, but no tetraploids as recorded by Devesa [52], and only tetraploid but no hexaploid *K. nevadensis*. In accordance with Devesa [52] we consider the report of 2*n* = 6*x* = 64 by Ehrendorfer [35] erroneous. In *K. dalmatica* we discovered only one tetraploid population, although previous data suggested diploidy [40]. Taking the cytotype diversity of several taxa into account, the failure to confirm previously recorded ploidy levels in the above-mentioned taxa is likely caused by the still limited number of investigated populations of these species. Alternatively, bearing in mind morphological variability and similarity of several species, some of the previously observed cytotypes might refer to different, misidentified species.

### Cytotype distribution patterns

Diploids and tetraploids are widespread throughout the distribution area of the genus (except for the eastern edge, where only diploids occur; Fig. 6), whereas hexaploids are restricted to the Alps (*K. dipsacifolia*, *K. illyrica* and *K. ressmannii*), the central Dinaric Mountains (*K. clementii*, *K. travnicensis* and *K.* sp. 1) and eastern Spain (*K. rupicola*). Hexaploid *K. dipsacifolia* occurs also north of the Alps [36], but those populations were not included in our study. Overall spatial patterns and phylogenetic data (Frajman *et al.*, unpubl. res.) suggest that tetraploids arose independently within almost all lineages of sect. *Trichera*, whereas hexaploids originated at least four times.

Among the widely distributed heteroploid species only *K. dinarica*, *K. drymeia* and *K. dipsacifolia*, and to some extent *K. arvensis*, exhibit geographic patterns of cytotype distributions. The northern populations of *K. dinarica* from the central Dinaric Mountains are diploid, whereas the populations in the southern Dinaric Mountains are tetraploid (Fig. 6). This is in accordance with Ehrendorfer [35], who, however, investigated only three populations. The contact zone between di- and tetraploids is approximately along the Sutjeska river valley, which was also identified as a major phylogeographic break in the *Heliosperma pusillum* group [53]. A similar pattern is observed on the Apennine Peninsula where the disjunct populations of *K. dinarica* subsp. *silana* from the southern Apennines are tetraploid, while the population from the central Apennines is diploid, suggesting connections with Balkan populations [54]. Amphiadriatic distributions are documented in several plant [55] and animal [56] species and are usually explained by land bridges across the Adriatic Sea during the Messinian salinity crisis (Miocene/Pliocene) or during Pleistocene glaciations [56, 57]. The diversification of sect. *Trichera* likely took place in the Pliocene/Pleistocene rendering Pleistocene migration more likely, but molecular dating analyses are needed to corroborate this hypothesis (Frajman *et al.*, unpubl. res.).

A similar amphiadriatic connection can be observed in *K. drymeia*. In this species diploids can be found only in the west (Italy and Slovenia) and east (Bulgaria, Macedonia, Romania, Serbia) of the distribution area, whereas tetraploids occur in intermittent areas (western Balkan Peninsula) and north of the diploids (Fig. 6). Interestingly, the isolated population from Gran Sasso in central Italy (from where the allegedly related *K. gussonei* Szabó was described) is tetraploid and AFLP data (Frajman *et al.*, unpubl. res.) suggest that it possibly results from transadriatic migration from the Balkan Peninsula.

Hexaploid *K. dipsacifolia* is limited to the Alps and the areas north of the Alps [36], whereas tetraploids occur in western Europe, the Carpathians and the Balkans (Fig. 6; [33, 42]). In *K. arvensis*, the species with the widest distribution area [36], our geographically limited sample showed that diploids are mostly distributed in the inner part of the Alpine arch, whereas tetraploids extend from its outer margin towards the northeast and southeast, with one population (K126) also in the Kvarner Bay of the northern Adriatic. The pattern in the northeast of the distribution area reflects the results of Kolář *et al.* [12], with the exception that we only included diploid populations from the Czech Republic.

### Genome size variation in Knautia: evolutionary considerations

Most of the results presented here are based on DAPI-stained nuclei (i.e., RGS), despite its preferential binding to AT-rich regions of DNA, because only dried material was available from most populations, which is not suitable for staining with the intercalating fluorochrome propidium iodide. However, as has been shown previously [58], the base content varies only slightly within a family and consequently, differences in fluorescence intensity of base-selective fluorochromes at low taxonomic levels (e.g., within a genus or a section) reflect variation in the total amount of nuclear DNA rather than variation in AT/GC base content [59]. Moreover, significant correlation of AGS and RGS values obtained from the same populations makes our conclusions based on RGS reliable.

The evolution of RGS shows strong correlation with the phylogeny of diploid members of *Knautia* (Fig. 5). *Knautia integrifolia* from sect. *Tricheroides* had the smallest monoploid RGS in the entire genus (Fig. 2), i.e. the 0.56-fold of the mean of sect. *Trichera,* which corroborates the results of Temsch and Greilhuber [45]. *Knautia integrifolia* was also exceptional in terms of intraspecific RGS variation amounting to 60 %, based on individual measurements (Fig. 2, Table 1). The species is distributed throughout much of the Mediterranean and in spite of our restricted sampling geography-correlated differences in RGS are manifested as the southern-most populations from Greece exhibited the smallest RGS while populations from Bulgaria, Italy and Croatia had larger RGS (Additional file 2: Table S1). Such variation in RGS is likely connected with wide distribution as well as phylogenetic heterogeneity, as the accessions of *K. integrifolia* are positioned in two or three well supported clades in ITS and plastid trees, respectively ([34], Fig. 5). Both investigated populations of *K.* cf. *degenii* have a similar RGS, on the lower limit of the variation exhibited by diploid members of sect. *Trichera. Knautia degenii*, for which no chromosome counts are available, was classified in sect. *Knautia* [36]. Phylogenetic analyses of ITS sequences ([34]; Fig. 5) showed its position within sect. *Tricheroides* and its GS is also closer to *K. integrifolia* than to *K. orientalis*. A gradual decrease in the RGS can be observed along the branch leading to sect. *Tricheroides* (Fig. 5). In contrast, *K. orientalis*, a member of sect. *Knautia*, has a RGS close to the maximum limit of the RGS variation of diploid members of sect. *Trichera* (Fig. 2) and, consequently, gradual increase in RGS can be observed along the branch leading to *K. orientalis* (Fig. 5). These results are in controversy with karyological data, as the base chromosome number *x* = 10 occurs in both sect. *Trichera* and *Tricheroides*, whereas *K. orientalis* from sect. *Knautia* has *x* = 8 ([36]; Fig. 5). Section *Knautia* is the earliest diverging lineage, whereas sect. *Tricheroides* is sister to sect. *Trichera* ([34]; Fig. 5); therefore more similar GS would be expected among diploids of the latter two lineages. Many factors, however, can influence the GS and the three lineages have likely diverged several million years ago, allowing for divergent GS evolution. Despite different chromosome numbers in sections *Knautia* and *Trichera* the GS of their diploid members is similar, whereas genome downsizing likely took place in the evolutionary history of sect. *Tricheroides*.

### Variation and geographic distribution of genome size in sect. Trichera

Within sect. *Trichera* RGS variation among diploids of different species was 1.69-fold, and among tetraploids 1.57-fold. The lowest level of variation (1.40-fold) was among hexaploids, which are also represented by the lowest number of species (and thus of analysed samples). Several Iberian taxa have higher GS: diploid *K. subscaposa* had an elevated RGS (Fig. 5) intermediate between diploids and tetraploids of other taxa, whereas tetraploid *K. legionensis*, *K. nevadensis* and *K. rupicola* exhibited RGS values intermediate between the remaining tetraploids and hexaploids, and hexaploid *K. rupicola* had the highest GS of all investigated taxa (Fig. 2). Accessions of *K. arvernensis* from central France and *K. lebrunii* from the eastern Pyrenees clustering together with the abovementioned taxa in AFLP analyses (Frajman *et al.*, unpubl. res.) had a smaller GS similar to the extra-Iberian members of sect. *Trichera*. Their similar GS, which is divergent from other taxa, provides additional evidence that the Iberian taxa are closely related, despite their different morphology (e.g., low-growing *K. rupicola* and *K. subscaposa* with strongly dissected leaves vs. tall-growing *K. nevadensis* with entire leaves; cf. [52]). Such GS increase was also observed in other plant groups, mostly connected with the amplification of certain classes of repetitive DNA, tandem repeats or transposable elements (*Vicia*, [60]; *Fritillaria*, [61]; *Melampodium* series *Leucantha*, [24, 49]; *Prospero*, [62]).

The present study revealed considerable intraspecific intra-ploidy level variation in RGS (Table 1), ranging from zero in tetraploid *K. foreziensis* endemic to a single mountain range to 1.21-fold in diploid *K. drymeia*, which has a much wider distribution. Also in *K. drymeia* and *K. integrifolia* (see above) high variation in RGS can be explained by evolutionary heterogeneity [34]. Diploid *K. drymeia* has a disjunct distribution, along the southern margin of the Alps (subsp. *centrifrons* (Borbás) Ehrend. and subsp. *tergestina* (G. Beck) Ehrend.) and in the southeastern Balkan Peninsula (subsp. *nympharum* (Boiss. & Heldr.) Ehrend.; Fig. 6b, [33]). Both groups are also genetically distinct (B. Frajman, unpublished AFLP data) and this could have triggered high variability in RGS. In general, the interpretation of such differences should be done with caution as several, especially widespread, *Knautia* species appear not to be monophyletic ([34], Frajman, unpubl. res.]). Also methodological artefacts can contribute to variability [10], but we consider the observed variation genuine from a methodological point of view. Analyses based on two different fluorochromes (PI and DAPI) gave consistent and correlated data, and simultaneous analyses of samples with different RGS yielded histograms with one bifurcate or two separate peaks (Fig. 4), which is considered the most convincing evidence for real differences in nuclear DNA content [10]. Moreover, similar differences in GS were observed in *K. arvensis* also by Kolář *et al.* [12]. The lack of chromosome counts for many analysed populations prevents explanation of the observed GS variation in terms of differences in the number of chromosomes (e.g. aneuploidy, dysploidy, B chromosomes). Nevertheless, intraspecific or even intrapopulation GS variation has been recently documented in several plant species [15, 63–67] indicating that genuine intraspecific variation in the size of the nuclear genome indeed exists.

Genome downsizing, i.e. a decrease in monoploid GS [18, 19] was observed in tetraploids and hexaploids of sect. *Trichera*. The monoploid RGS of tetraploids and hexaploids was significantly smaller than that of diploids, whereas there was no significant difference between tetraploids and hexaploids (Fig. 3). Genome downsizing following polyploidisation has been reported for other taxa and appears to be more common than genome expansion [19]. It is usually explained by elimination of repetitive or single copy DNA sequences [9, 19, 20, 68–70]. An alternative, yet untestable, hypothesis is that the parental species had smaller GS when they gave rise to a polyploid derivative. The extent of genome downsizing is group-specific but in general more pronounced in old polyploids [23, 48, 71–74].

The spatial distribution of monoploid RGS in sect. *Trichera* was non-random. Within di- and tetraploid cytotypes smaller monoploid RGS values were observed in the western Balkan Peninsula and the Alps. A weak, albeit statistically significant increase of monoploid RGS was observed towards the eastern, western and southern margins of the section’s distribution in diploids and towards the western and northern margins in tetraploids and hexaploids (Fig. 7). A similar pattern of smaller monoploid RGS in the distribution centre, and an increase towards the margins of the range were also observed in the grass genus *Sesleria*, with centre of diversity in the Balkan Peninsula [75]. We are not aware, however, of other studies showing such pattern, probably also because most studies evaluating GS across multiple species of a genus did not cover their geographic distribution or did not explore the geographic distribution of monoploid GS. Future experimental studies will show whether larger GS in *Knautia* limits adaptive and competitive abilities of populations [76] at the genus’ distribution margins and might thus represent a factor limiting further range expansion. Genome size increase is typically associated with polyploidisation or increased activity of retrotransposons [77, 78], which is often caused by stress factors, both genomic and environmental [79, 80]. The larger genome sizes of *Knautia* taxa at the distribution margins might, thus, be caused by activation of certain types of retrotransposons in response to stress triggered by colonization of new habitats. Alternatively, taxa with larger genomes might have been better suited to occupy new niches.

## Methods

### Plant material

Plant material for GS and chromosome number estimation was collected in the field in 2010–2013 throughout the distribution range of the genus. For RGS measurements leaf material of one to ten individuals per population and one to 65 populations per species (i.e., roughly proportional to the size of the species’ distribution areas) was collected and immediately desiccated in silica gel. Plants collected in the field were grown in the Botanical Garden of the University of Innsbruck and plants were used for AGS measurements and karyology.

Plant species determinations are based on Flora Europaea [33] and national floras [52, 81–83]. Herbarium vouchers were revised by F. Ehrendorfer, a taxonomic expert of the group. Five populations could not be assigned to any taxon, four of which were hexaploid from Velebit in Croatia (*K.* sp. 1: K102, K103, K105, K500) and one was a tetraploid from Serbia (*K.* sp. 2: K218).

Voucher specimens are deposited either at the Institute of Botany, University of Innsbruck, Austria (IB), the Faculty of Science, University of Zagreb, Croatia (ZA), the Faculty of Agriculture, University of Zagreb, Croatia (ZAGR), the Faculty of Biology, University of Belgrade, Serbia (BEOU) or the Natural History Museum Belgrade, Serbia (BEO; Table S1). Voucher numbers, collection details and taxonomic authorities are given in the Additional file 2: Table S1; further information on the populations can be retrieved from the publicly accessible database of the BALKBIODIV project at http://www.uibk.ac.at/botany/balkbiodiv/?Sampling_sites.

### Chromosome numbers

Chromosome numbers were determined for 14 *Knautia* species (Table 1). Root tip meristems were pre-treated with 0.002 M aqueous solution of 8-hydroxyquinoline for 2 h at room temperature and 2 h at 4 °C, fixed in 96 % ethanol and glacial acetic acid (3 : 1), and stored at -20 °C until use. Meristems were hydrolysed in 5 N HCl for 30 min at room temperature, washed with tap water and stained with Schiff’s reagent for one hour [84]. Chromosome spreads were prepared by squashing a stained meristem in a drop of acetic acid (60 %) under the coverslip, and analysed using AxioImager M2 microscope (Carl Zeiss, Vienna, Austria). Images were acquired with a CCD camera and files processed using AxioVision ver. 4.8 (Carl Zeiss, Vienna, Austria). Chromosomes were cut out in Corel PHOTO-PAINT X5 and used for constructing karyotypes.

### Flow cytometry

Flow cytometry (FCM) of 4’,6-diamidino-2-phenylindole (DAPI; final concentration 0.036 M) stained nuclei was used to estimate RGS and to assess DNA ploidy levels of silica gel-dried *Knautia* samples [85]. The primary internal standard used to determine DNA amounts was *Vicia faba* cv. Inovec (2C = 26.90 pg; [86]). For the samples K401 and K402 of *K. rupicola* the internal standard was *Pisum sativum* cv. Kleine Rheinländerin (2C = 8.84 pg; [87]) as the sample peak overlapped with that of *Vicia faba*. Desiccated green leaf tissue (c. 0.5 cm^2^) of two individuals from the same population were pooled together with an appropriate amount of fresh reference standard and processed as described in Suda *et al.* [15]. The relative fluorescence intensity of 3,000 particles was recorded using a Partec CyFlow Space flow cytometer (Partec GmbH, Münster, Germany). Partec FloMax software was used to evaluate the histograms, which were manually gated. RGS was calculated as ratio between the relative fluorescence of sample and standard. The reliability of the measurements was assessed by calculating coefficients of variation (CV) for the G_1_ peaks of both the analysed sample and the reference standard. Analyses yielding a CV threshold of > 5 % were discarded and the samples measured again. The number of measurements per population yielding high quality FCM histograms is given in Table 1.

AGS was determined using FCM of propidium iodide (PI) stained nuclei of 88 samples from 30 species (see Table 1), covering the entire GS variation encountered in RGS measurements. Fresh, intact leaf tissue was co-chopped with leaf material of the reference standard *V. faba* cv. Inovec (2C = 26.90 pg; [86]) in Otto I buffer (0.1 M citric acid monohydrate with 0.5 % Tween 20; [88]). For *K. rupicola* K402 the standard was *Secale cereale* cv. Dankovská (2C = 16.19 pg; [89]), as the peak of this accession was overlapping with the peak of *V. faba*. Otto II buffer (0.4 M Na_2_HPO_4_) with β-mercaptoethanol (2 μl/ml), RNase (50 μg/ml) and PI (50 μg/ml) was added to the flow-through fraction and incubated for 5–15 min. The relative fluorescence intensity of 5,000 particles was recorded. The AGS of each sample was measured three times on different days in order to eliminate system errors (the AGS of each sample is thus a mean value of three measurements). Therefore, aliquots of nuclear suspensions were preserved in 500 μl glycerol (60 %) at -20 °C as described in Kolář *et al.* [90]. Prior to FCM analysis, the suspension was centrifuged (3 min at 3,200 rpm), the supernatant was discarded and the nuclei were resuspended in 200 μl of Otto I buffer. After incubation for 15 min at room temperature, the samples were stained with PI, incubated for 5–15 min and their GS was measured. A divergence of up to 2 % between the three measurements and a CV of up to 3 % was accepted.

### Statistical analyses

Due to the non-normal distribution of RGS within ploidy levels (as assessed by Kolmogorov-Smirnov test) Spearman rank order correlations implemented in Statistica 12 (StatSoft Inc., Tulsa, OK, USA) were calculated to assess the relationship between RGS and AGS across all three ploidy levels and for each ploidy level separately, as well as between ploidy level (i.e., number of complete chromosome sets) and monoploid RGS. The same analysis was used to test the relationships between monoploid RGS of a population and the geographical distance of that population from an arbitrarily defined coordinate positioned in an area with high frequency of small monoploid RGS on the western Balkan Peninsula (43° 30‘N, 18° 00‘E; distances determined with ArcGIS 10, ESRI). The latter analyses were performed for the entire sect. *Trichera* as well as for each ploidy level separately. Finally, Kruskal-Wallis tests followed by Mann–Whitney pairwise comparisons and Bonferroni correction of p values conducted with PAST 3 [91] were used to test for differences in RGS and monoploid RGS among ploidy levels within sect. *Trichera*.

### Phylogenetic analyses and genome size reconstruction

Evolution of RGS was reconstructed for diploid taxa only, for which sequences were available from the study of Rešetnik *et al.* [34]. For *K.* sect. *Knautia* and *K.* sect. *Tricheroides* ITS and plastid *petN*(*ycf6*-*psbM*) datasets from Rešetnik *et al.* [34] were pruned to contain all accessions for which RGS data were available; the putative hybrid *K. degenii* K272 was excluded. For *K.* sect. *Trichera* only two to three accessions with smallest and highest RGS per species were retained covering the entire intraspecific RGS variation. Bayesian analyses were performed for the concatenated dataset employing MrBayes 3.2.1 [92] applying the GTR + Γ substitution model proposed by the Akaike information criterion implemented in MrAIC.pl 1.4 [93] for both datasets. The settings for the Metropolis-coupled Markov chain Monte Carlo (MC3) process included four runs with four chains each (three heated ones using the default heating scheme), run simultaneously for 10,000,000 generations each, sampling trees every 1,000th generation using default priors. Posterior probabilities (PPs) were determined from the combined set of trees, discarding the first 1001 trees of each run as burn-in. The evolution of RGS was mapped onto the Bayesian consensus phylogram applying the R package [94] phytools (methods contMap and phylosig; [95, 96]). We used phylosig and the implemented Chi-square test to test for the presence of phylogenetic signal, i.e. to determine whether the distribution of RGS is properly predicted by our phylogenetic hypothesis (λ > 0) or distributed randomly (λ = 0).

## Conclusions

We confirmed the presence of three ploidy levels (diploids, tetraploids and hexaploids) in *Knautia*; diploids and tetraploids are widespread whereas hexaploids have a more restricted distribution. Several species are heteroploid, and in some species cytotypes exhibit geography-correlated distribution patterns. Altogether, we show that the application of flow cytometry and chromosome counts to a comprehensive taxonomic and dense geographical sampling enables the recognition of evolutionary patterns such as the strongly deviating genome size of Iberian taxa or the centrifugal increase in genome size across ploidy levels towards the range limits of section *Trichera*. Further phylogeographic, phylogenetic, cytological and ecological analyses will allow elucidating causes underlying the observed patterns.

### Availability of supporting data

The data sets supporting the results of this article are included within the article and its additional files.

## Additional files

Additional file 1: Figure S1. Karyotypes of three diploid, two tetraploid and one hexaploid species of *Knautia*. Scale bar equals 5 μm. Additional file 2: Table S1. Voucher information and genome size data. More information can be retrieved from http://www.uibk.ac.at/botany/balkbiodiv/?BALKBIODIV under “Sampling sites”.
