# Supplementary figures and images for: Cytotype diversity and genome size variation in Knautia (Caprifoliaceae, Dipsacoideae)
Source: BMC Evol Biol. 2015 Jul 17;15:140. doi: 10.1186/s12862-015-0425-y (PMC4504173; doi:10.1186/s12862-015-0425-y)

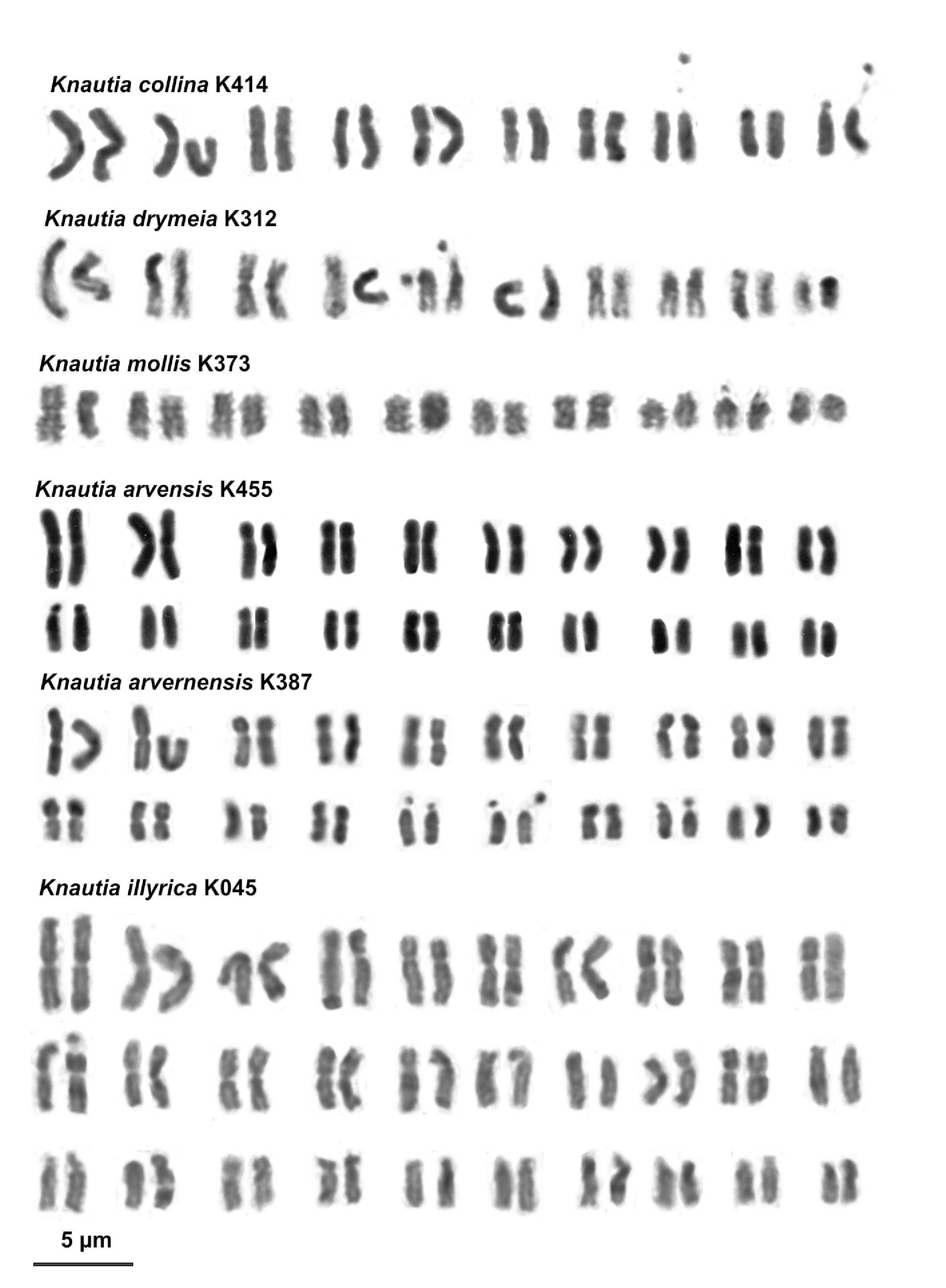

Supplement: Additional file 1: Figure S1. — Karyotypes of three diploid, two tetraploid and one hexaploid species of Knautia. Scale bar equals 5 μm. [file 12862_2015_425_MOESM1_ESM.jpg]
